# Attention can shift the reference eye under binocular fusion failure: A case report

**DOI:** 10.1167/jov.25.14.15

**Published:** 2025-12-17

**Authors:** Jiahao Wu, Tengfei Han, Qian Wang, Lian Tang, Yumei Zhang, Zhanjun Zhang, Zaizhu Han

**Affiliations:** 1 National Key Laboratory of Cognitive Neuroscience and Learning & IDG/McGovern Institute for Brain Research, Beijing Normal University, Beijing, China; 2 Innovation Institute of Integrated Traditional Chinese and Western Medicine, Shandong First Medical University & Shandong Academy of Medical Sciences, Jinan, Shandong, China; 3 Department of Rehabilitation, Beijing Tiantan Hospital, Capital Medical University, Beijing, China

**Keywords:** binocular fusion, reference eye, cyclopean eye, cerebellar vermis, single case report

## Abstract

Binocular fusion normally relies on a “cyclopean eye” that integrates image disparities between the two eyes into a single coherent percept. When fusion fails, how the brain chooses its spatial reference frame remains unclear. Here, we report a rare case of a 44-year-old man who developed multiple-directions diplopia following surgical resection of a cerebellar vermis hemangioblastoma. Clinical tests showed deficits in several extraocular muscles. Experimentally, in binocular and dichoptic viewing, perception was always anchored to the left eye with the right eye's image misaligned, whereas monocular viewing produced no diplopia. Crucially, the patient could voluntarily switch to the right eye as reference, which was independent of stimulus shape similarity, stimulus exposure order, or participant response demands. This case offers a unique window to understand the relationship between automatic sensory integration and top-down control in binocular vision: When cyclopean fusion breaks down, visual perception adapts to a single-eye reference frame that can be flexibly influenced by attention.

## Introduction

A long evolutionary process in living species has led to the development of bilaterally symmetrical sense organs (e.g., eyes, ears, and nostrils). This bilateralism provides significant advantages for survival and reproduction. For instance, having two eyes rather than one enables faster recognition of visual stimuli, sharper vision, a wider field of view, and depth perception. A critically relevant scientific question is how the brain integrates information from two separate eyes into a single, coherent image (i.e., binocular fusion).

Research on healthy individuals has shown that visual information from both eyes is fused to form a virtual “cyclopean eye,” located midway between the two physical eyes, resulting in a unified percept (Barendregt, Harvey, Rokers, & Dumoulin, 2015; Cox, Dougherty, Westerberg, Schall, & Maier, 2019; Mapp, Ono, & Barbeito, 2003; Ono & Barbeito, 1982; Radhakrishnan, Dorronsoro, Sawides, Webster, & Marcos, 2015; Welchman, 2016; Whritner, Czuba, Cormack, & Huk, 2021). However, certain disorders caused by dysfunction of the extraocular muscles—such as strabismus, diplopia, and alternating exotropia—disrupt binocular fusion. In such cases, the spatial alignment of visual input from one or both eyes is disturbed, leading to the perception of two separate images instead of a single coherent one (Benhaim‐Sitbon, Lev, & Polat, 2022; Jampel, 1955; Leigh & Zee, 2015; Rutstein, 2017; Sharpe, 1979). This raises two particularly interesting questions when binocular fusion is disrupted: (a) What is the “reference eye”—that is, the eye whose visual input provides the spatial anchor determining which image appears upright and stable in perception? Is it still the virtual geometric midpoint (the cyclopean eye) or one of the physical eyes? (b) If the reference eye is one of the physical eyes, can it shift between the two eyes under the influence of high-level attention? Previous reports have struggled to answer these questions. This is simply because most cases involve damage to a single extraocular muscle, resulting in limited vertical or horizontal image displacement (Kumar, Kaur, Raj, Lal, & Sukhija, 2021; Merino, Cerdán Llach, Gago Argüello, Gómez de Liaño, & Yáñez-Merino, 2024). The extent of misalignment in such cases is often too small to clearly identify the reference eye.

Fortunately, we recruited a case with strabismus involving dysfunction in multiple extraocular muscles, resulting in pronounced vertical, horizontal, and torsional (tilted) image displacement. This case offers a rare opportunity to investigate the nature and flexibility of the reference eye.

## Patient presentation

**Figure 1.**
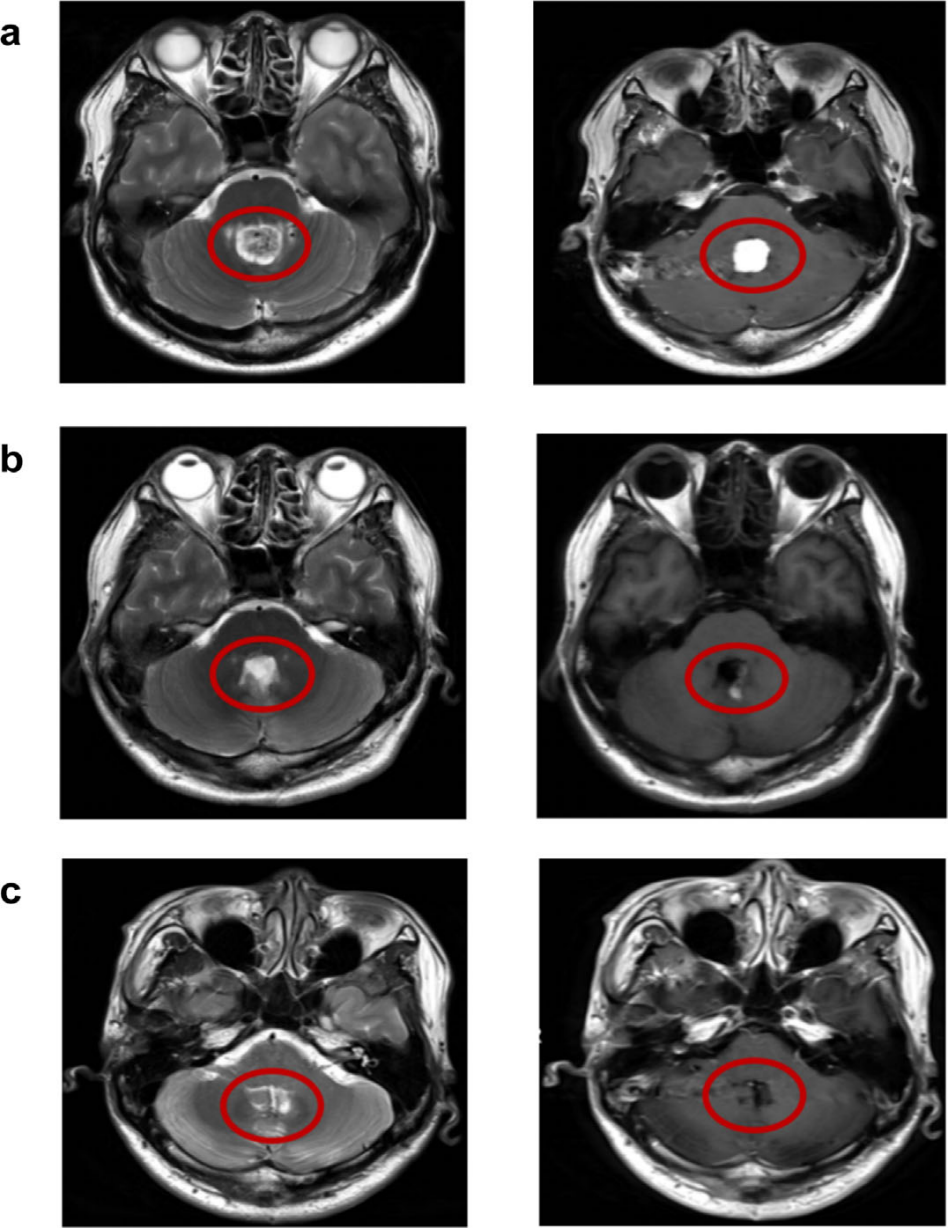
Pre- and postoperative brain magnetic resonance imaging scans of the patient. (**a**) Preoperative hemangioblastoma in the fourth ventricle/vermis. (**b**) One week after resection, marked edema. (**c**) Three months after resection. In each panel, the left image is T2-weighted, and the right image is T1-weighted. The red circle indicates the hypointense signal in the midline cerebellar vermis.

This patient was a 44-year-old male engineer with a bachelor's degree. Prior to October 2024, he was in generally good health, aside from bilateral high myopia and mild astigmatism (Table 1). In October 2024, he began experiencing dizziness and gait instability and was subsequently admitted to Beijing Tiantan Hospital. A space-occupying lesion involving the fourth ventricle and cerebellar vermis was identified (Figure 1a) and diagnosed as a hemangioblastoma. Surgical resection of the lesion was performed in January 2025. One week after the surgery, the resected region exhibited significant edema, which had markedly subsided by 3 months postoperatively (Figures 1b, 1c). Subsequently, most of his postoperative symptoms had resolved, except for persistent diplopia.

**Table 1.** Pre- and postoperative refraction test results of the patient. *Notes*: Spherical power (in diopters, D) indicates the degree of myopia (negative values) or hyperopia (positive values); cylindrical power (D) denotes the magnitude of astigmatism; axis (in degrees) specifies the orientation of the cylindrical lens meridian. Pre and post columns compare measurements taken before and after the surgical resection, respectively.

	Spherical (D)		Cylindrical (D)		Axis (°)	
	Pre	Post	Pre	Post	Pre	Post
Right	−7.00	−7.00	0.25	−1.75	91	78
Left	−6.75	−7.50	−0.50	−1.75	112	101

## Experimental procedure

### Ophthalmic tests

We first administered the following standard clinical ophthalmic tests to assess the patient's refractive error and binocular alignment.

#### Refraction test

This test is used to examine the refractive status of the eye, including the degree of myopia, hyperopia, and astigmatism (Dandona & Dandona, 2001; Jin et al., 2015; Leone, Mitchell, Morgan, Kifley, & Rose, 2010). It revealed that the patient had consistently high myopia in both eyes before and after surgery (December 2024 and April 2025), accompanied by a notable postoperative increase in astigmatism (Table 1).

#### Hess screen test

The Hess test can be relatively accurate at mapping the magnitude of misalignment of extraocular muscles in different positions of gaze (Armesto, Ugrin, Travelletti, Schlaen, & Piantanida, 2008; Lancaster, 1939; Quaid & Hamilton-Wright, 2010). During the test, the patient viewed a grid of fixation targets through red–blue filters that dissociated the two eyes. One eye (the fixing eye) was presented with a central reference target, while the other eye (the tested eye) viewed a movable target. The patient was instructed to align the two targets using a mouse-controlled cursor. This procedure was repeated across multiple gaze directions covering the horizontal, vertical, and oblique fields. Each alignment response generated a point on the Hess plot, reflecting the relative deviation of the tested eye with respect to the fixing eye. The resulting projection provided a two-dimensional map of ocular deviation, known as the Hess plot.

**Figure 2.**
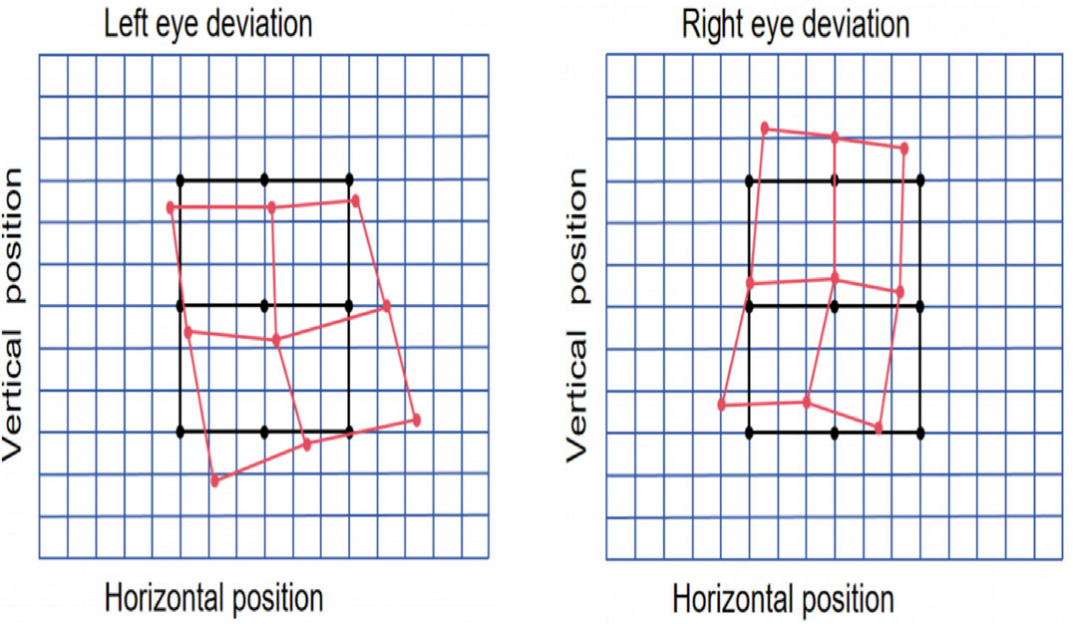
Hess test results of the patient. Black traces denote the normal reference frame, and red traces show the patient's gaze positions across nine directions of fixation.

Figure 2 shows the patient's Hess plots of each eye. The black grid represents the normal reference positions, while the red grid indicates the patient's actual ocular deviations. When the left eye was tested (right eye fixed), the plotted field shifted toward the nasal visual field and slightly downward, with a mild clockwise tilt, suggesting weakness of the left lateral rectus and minor vertical muscle involvement. When the right eye was tested (left eye fixed), a similar nasal and downward displacement with tilt was observed, indicating dysfunction of the right lateral rectus and associated vertical muscles.

### Tests of binocular, monocular, and dichoptic viewing

**Figure 3.**
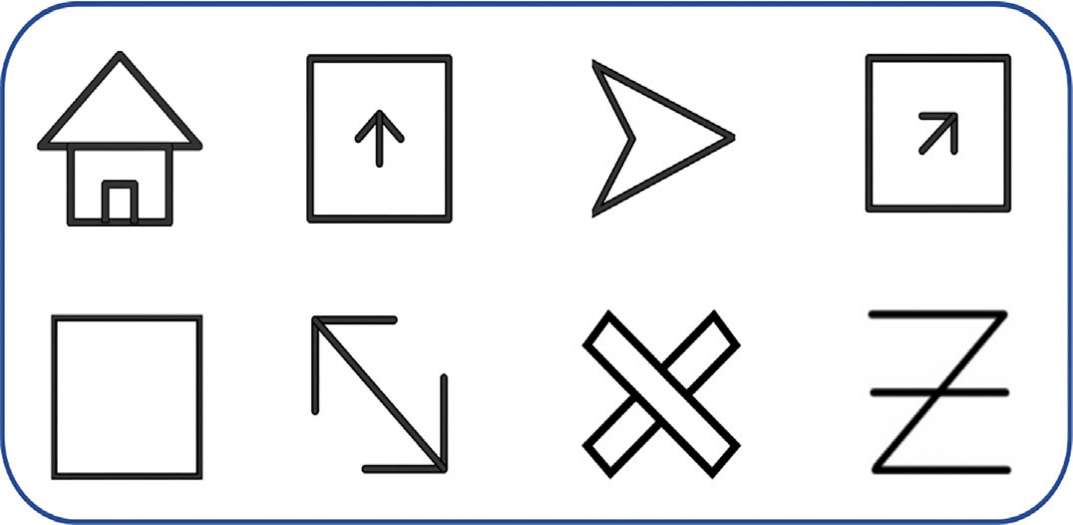
The eight standardized visual stimuli used across all tests.

Eight representative visual stimuli were used. Each stimulus was a high-contrast black-and-white line drawing (luminance: 0.2 cd/m²) (Figure 3). The images were simple, clear, and had distinct orientations. Three images exhibited diagonal symmetry, three showed either horizontal or vertical symmetry, one was fully symmetrical, and one was asymmetrical.

#### Binocular viewing

**Figure 4.**
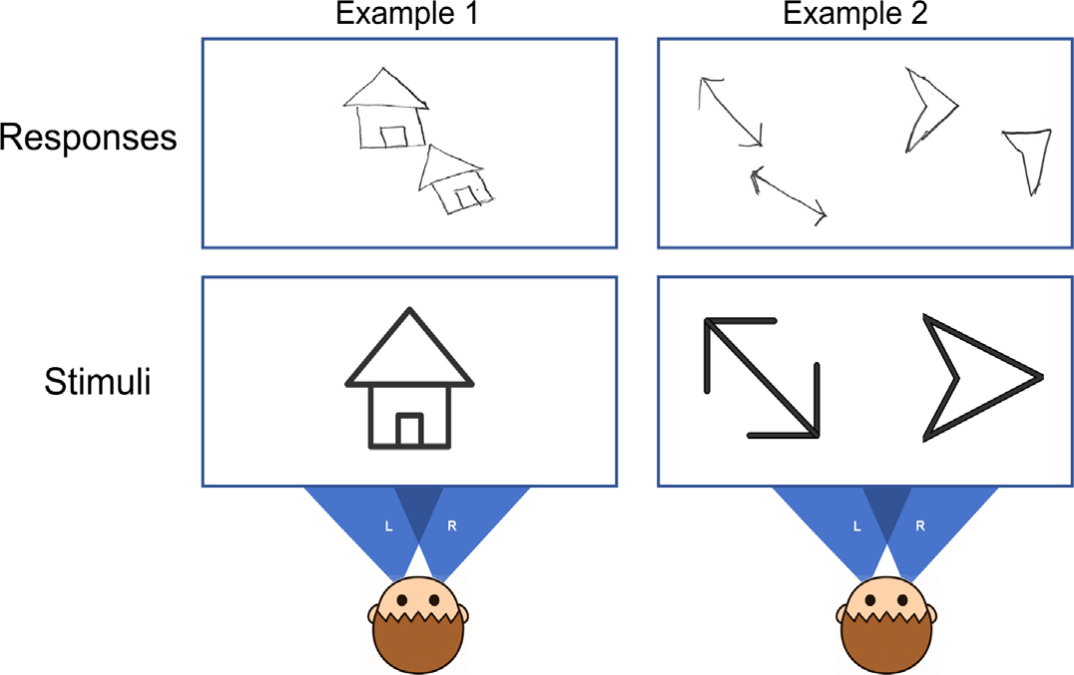
Example responses of the patient under the binocular viewing condition. In Example 1, the patient was presented with a single stimulus; in Example 2, with two stimuli. The shaded areas, labeled “L” and “R,” illustrate the patient's left and right visual fields, respectively.

Each stimulus was presented directly in front of the patient at a distance of 30 cm, with his head stabilized using a chin rest. The stimuli were printed on white A4-sized paper (210 × 297 mm). The patient viewed the stimuli naturally with both eyes and subsequently drew his perceptual impressions on a separate blank sheet of the same size. There were no time constraints for either viewing or drawing. A total of 16 trials were conducted. The patient exhibited a complex pattern of binocular disparity, characterized by mild horizontal (26 ± 18.48 mm) and vertical (25 ± 13.15 mm) offsets, along with a relatively large angular deviation (28 ± 8.05°). These measurements were referenced to the left-eye image, with positive values indicating upward, rightward, and clockwise shifts (see an example in Figure 4). In brief, the patient exhibited a failure of binocular fusion, perceiving a single visual target figure as two separate figures with different directions and positions—one matching the target in both orientation and location, and the other displaced both translationally and rotationally relative to the target.

#### Monocular viewing

**Figure 5.**
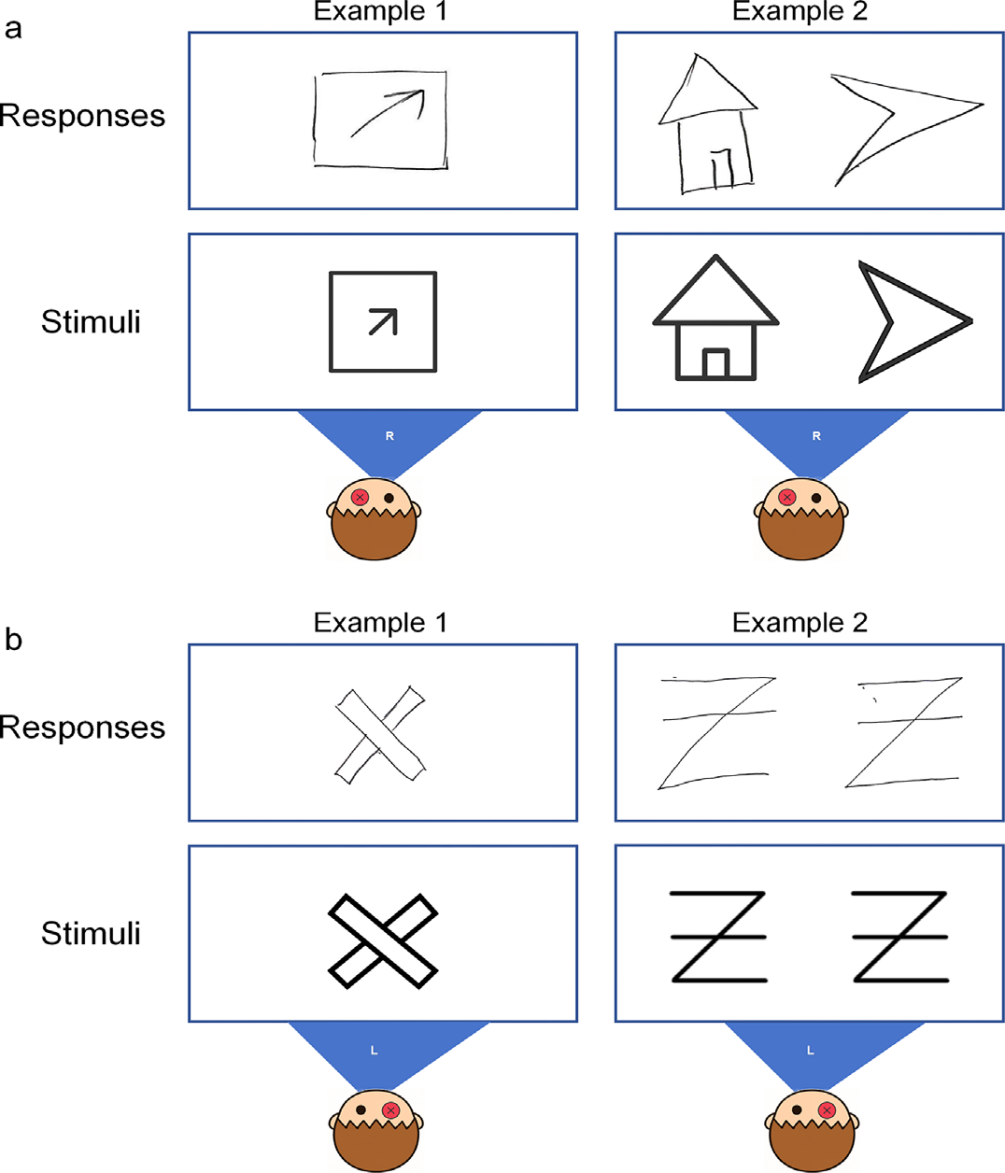
Two representative drawings from the patient in the monocular viewing condition, with the single-item trials shown in the left column and the two-item trials in the right column. (**a**) Drawings produced when viewing with the left eye only. (**b**) Drawings produced when viewing with the right eye only.

The testing procedure was identical to that used in the binocular viewing condition, except that one of the patient's eyes was covered with an eye patch, allowing for monocular viewing. The patient was explicitly instructed to align the drawn stimulus relative to the paper's edges and to ensure that the perceived upright orientation matched the paper's vertical axis. A total of 16 trials (8 trials for each eye) were conducted. The patient was able to accurately reproduce all stimuli without experiencing diplopia or image translation and rotation (see examples in Figure 5), suggesting that his monocular visual function was well preserved.

#### Dichoptic viewing

The testing procedure was identical to that used in the binocular viewing condition, except that a haploscope was employed to separate the visual fields of the two eyes, allowing each eye to view independently, thereby eliminating binocular fusion. In each trial, two stimuli were presented simultaneously, with each eye viewing one stimulus. The stimuli shown to the left and right eyes were randomly selected from a set of eight. A total of 16 trials were conducted. He again exhibited the binocular disparity similar to that observed in the binocular viewing condition above a relatively large angular deviation (25.3 ± 6.95°).

**Figure 6.**
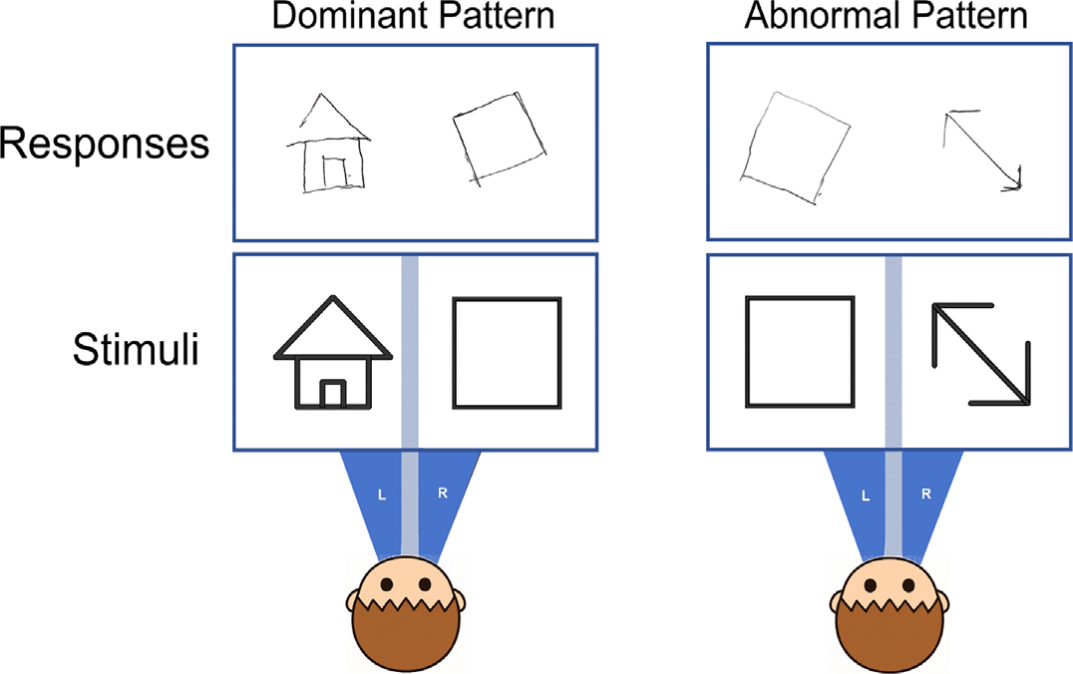
A representative drawing of the patient from the dichoptic viewing condition.

A novel and unexpected finding emerged: In 12 of the 16 drawings, the image in the left visual field was aligned with the stimulus, while the image in the right visual field exhibited rotational deviation. In contrast, the remaining four drawings showed the opposite pattern—alignment in the right visual field and deviation in the left (see an example in Figure 6). Additionally, he subjectively reported that he could voluntarily switch his reference eye during the test. These results suggest that he was capable of shifting the “reference eye” between the left and right eyes during dichoptic viewing.

To further investigate the underlying mechanism of his reference-eye shift during dichoptic viewing, we conducted a series of additional tests. We were particularly interested in identifying the initial reference eye (left or right) and assessing the extent to which he could voluntarily shift his reference eye through high-level intentional control.

### Subjective shift of reference eye during dichoptic viewing

#### Identifying the initial reference eye

**Figure 7.**
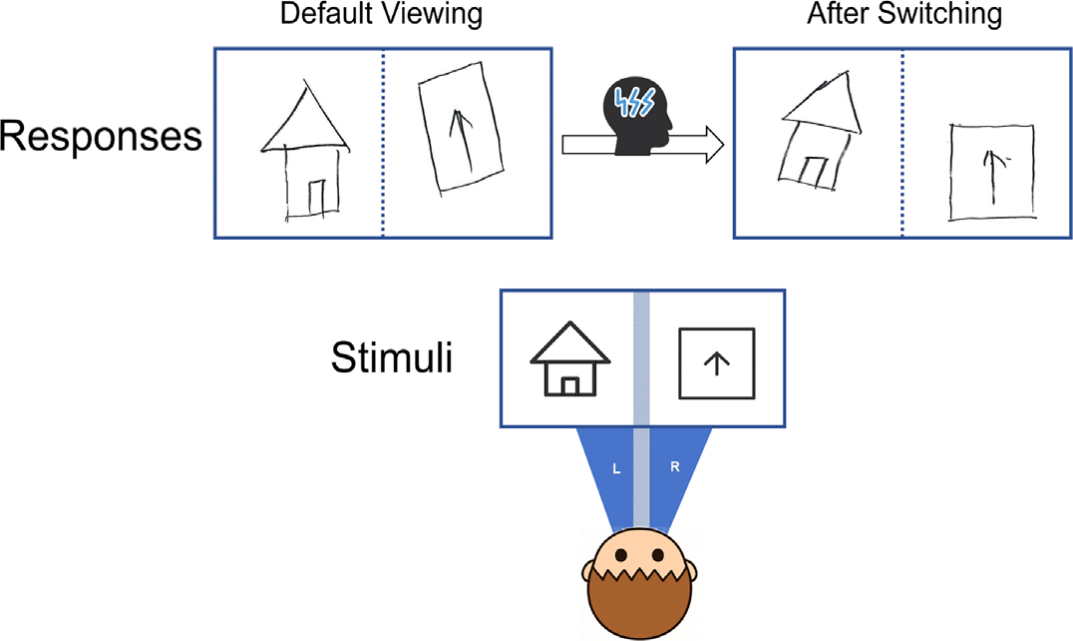
Representative drawings from reference-eye switching of the patient.

This testing procedure was identical to the previous dichoptic viewing test, except that the response was divided into two steps rather than one. In the first step, the patient was instructed to draw the perceived stimuli based on his initial visual impression. In the second step, he was asked to subjectively switch his reference eye. Rather than using this technical term, the experimenter instructed the patient to make the image from the initially tilted eye appear upright and stable (e.g., “Please adjust so that the image seen by your left/right eye looks upright and steady.”). If he was able to successfully shift the reference eye, he drew the newly perceived stimuli; otherwise, he provided a verbal report. In the first step, across all 16 trials, the patient perceived the left visual-field images as correctly aligned, while the right-field images appeared rotated (19.96 ± 3.96°). In the second step, in all the trials but one, he successfully shifted his reference eye, producing a reversed pattern—misalignment in the left visual field (20.97 ± 8.69°) and correctly aligned images in the right visual field (see an example in Figure 7). These results indicate that this patient's default reference eye during dichoptic viewing was the left eye, but he could voluntarily switch to the right eye through high-level intentional control.

#### Investigating the influence of stimulus similarity

**Figure 8.**
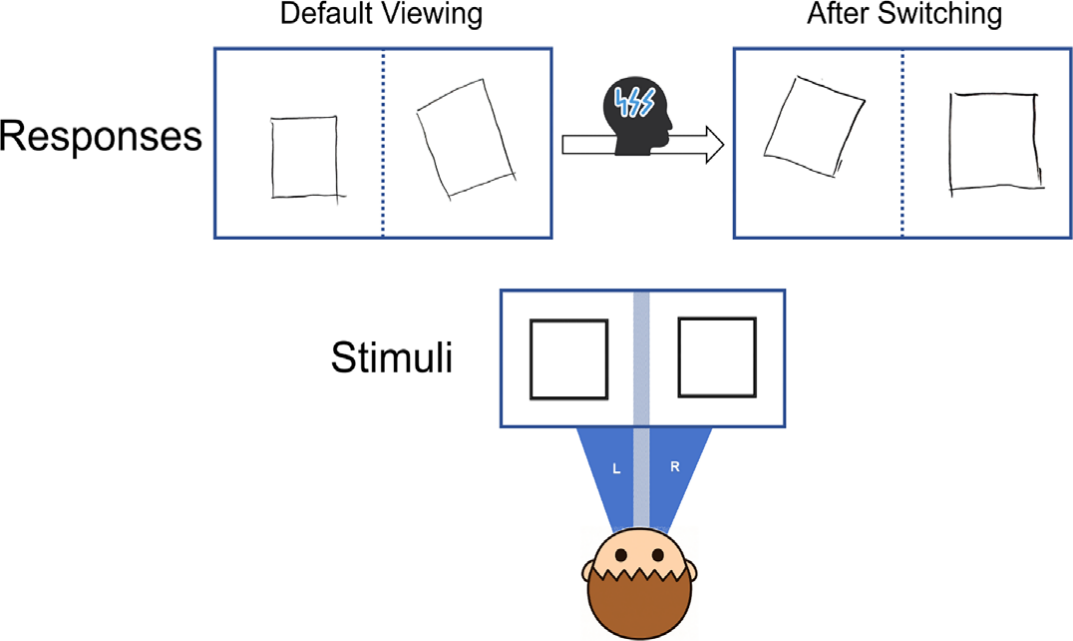
Representative drawings of the patient from reference-eye switching for the same stimulus.

In the previous task, each trial involved two different stimuli, with each eye viewing a distinct image. This raises an interesting question: Would his performance pattern change if both eyes were presented with the same stimulus? To explore this, we asked him to repeat the test, but this time with identical stimuli presented to both eyes. Each of the eight figures was used in one trial. We found that even when the stimuli were the same, he consistently used the left eye as the initial reference eye in all eight trials (deviation angle degree of right field: 21.45 ± 4.38°). Moreover, he was able to voluntarily modulate the reference to the right eye for all eight trials (deviation angle degree of left field: 22.43 ± 9.13°) (see an example in Figure 8).

#### Investigating the influence of eye exposure order

**Figure 9.**
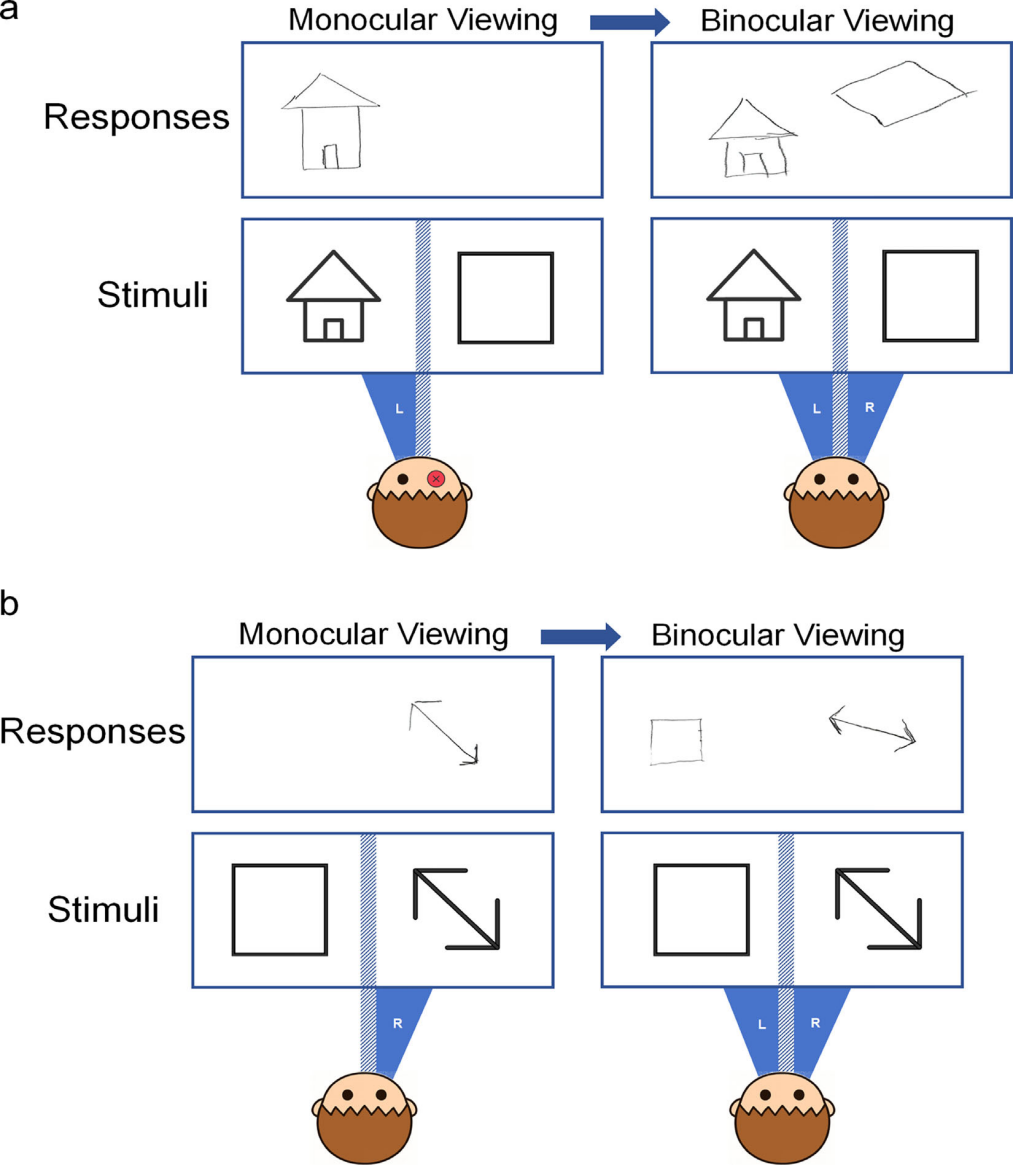
Representative drawings from the patient in the reference-eye switching condition, illustrating different eye exposure orders. (**a**) Drawings produced when viewing first with the left eye and then with both eyes. (**b**) Drawings produced when viewing first with the right eye and then with both eyes.

To further examine whether the reference-eye pattern would change when the two stimuli were viewed sequentially rather than simultaneously, we modified the task to present the images to each eye in a specific order. This patient once again performed the “Identifying the Initial Reference Eye” task, with the following adjustment. In each trial, he first viewed the stimulus for 1 second with only one eye open (either the left or right eye) while the other was closed. Then, the second eye was opened, allowing binocular viewing. We found that, regardless of whether the left or right eye viewed the stimulus first, the left eye consistently served as the initial reference eye for him when perceiving tilted images in the right visual field (deviation angle degree: 25.04 ± 2.53°) (see an example in Figure 9). These results suggest that the order of eye exposure had no influence on the reference-eye selection during subsequent binocular viewing.

#### Examining the influence of response requirements

In the previous tasks, responses were made by manually drawing the perceived stimuli—a method that was time-consuming and imposed a considerable physical burden on him. This raises an important question: Would he exhibit the same performance pattern under conditions with reduced response demands? To investigate this, we asked him to repeat the task, with the only modification being the response method—replacing manual drawing with key presses. The same visual stimulus configuration as in the preceding drawing tasks was used, with identical orientations and spatial arrangements. In the first step, he pressed the left (**←**) or right (**→**) arrow key to indicate whether the tilted image appeared in the left or right eye, respectively. In the second step, he pressed either the “YES” or “NO” key to report whether he was able to successfully shift the reference eye. In the first step, he perceived all 50 trials as originating from the left reference eye, with an average response time of 2.13 seconds (*SD* = 0.86 seconds; range = 1.04–4.53 seconds). In the second step, he successfully shifted the reference eye in 41 of 50 trials, with an average response time of 13.33 seconds (*SD* = 10.81 seconds; range = 1.23–55.37 seconds). These results demonstrate that his pattern of subjective reference-eye shifting was preserved even under a low-burden response condition.

## Discussion

According to the “cyclopean eye” theory, a unified, midpoint reference emerges when disparities of two eyes lie within fusional limits (Panum area). In this study, the patient's large horizontal, vertical, and torsional deviations made cyclopean alignment impossible, forcing reliance on a single eye frame. Although psychophysical and clinical reports have long noted that strabismic or alternating exotropia patients tend to use one eye as their anchor (Economides, Adams, & Horton, 2014; Mapp et al., 2003), this study offers the first clear and documented evidence of this strategy through systematic tasks.

The ability to voluntarily switch reference eyes under failure of binocular fusion reveals that reference-eye choice is not a purely low-level, automatic process but could be directed by attention. To our knowledge, a systematic demonstration of volitional reference-eye switching in such a case of fusion failure has not been reported, but it is consistent with many theoretical models and clinical observations (Marx & Einhauser 2015; Song, Lyu, Zhao, & Bao, 2023; Treisman, 1969; Wang, McGraw, & Ledgeway, 2021; Zhang, Jiang, & He, 2012). Specifically, neurophysiological models of binocular gain control (Ding & Sperling, 2006; Ding & Sperling, 2021) propose that binocular fusion is not a simple summation but rather a weighted combination at the V1 level, where each eye's input is scaled by a dynamic gain factor. In our patient, intentionally anchoring to the right eye from the left eye would elevate its cortical weight, thereby deciding the right-eye image as the spatial reference. A historical neuropsychological study (Schaadt, Brandt, Kraft, & Kerkhoff, 2015) also implicated higher cortical regions in sustaining stable fusion, reporting that impaired binocular fusion of “flat vision” was led by right parietal damage. Furthermore, numerous studies have shown that attention allocation can dynamically modulate sensory eye dominance (Dieter, Sy, & Blake, 2017; Johansson, Seimyr, & Pansell, 2015; Khan & Crawford, 2001; Mitchell, Carlson, Westerberg, Cox, & Maier, 2023). These findings all resonate with our results, indicating that binocular fusion and perceptual weighting are not strictly automatic; rather, higher-order processes can flexibly intervene when needed.

In this case, we suggest that the patient's capacity for voluntary reference-eye selection arises from disruption of the normally automated anchoring mechanisms. The patient's surgery damaged the cerebellum vermis—a region increasingly recognized for its role in visuospatial processing and eye movement calibration (Kheradmand & Zee, 2011; Nitta, Akao, Kurkin, & Fukushima, 2008a; Nitta, Akao, Kurkin, & Fukushima, 2008b; Takagi, Tamargo & Zee, 2003; Takagi, Zee, & Tamargo, 1998; Van Es, Van Der Zwaag, & Knapen, 2019; Versino, Cosi, D'Oria, & Colnaghi, 1996). We therefore propose that a healthy cerebellar-vermis-linked network may automatically determine whether a unified cyclopean reference or a single-eye frame should anchor perception, without requiring conscious effort. In the patient, damage to his vermis region may have disrupted this automation, which makes the brain have to recruit attention to select the reference eye consciously.

This capacity for voluntary reference-eye selection, once the cerebellar “autopilot” is off, can be viewed as an instance of attentional selection-for-perception, complementing the selection-for-action effects reported by Economides et al. (2014). Within the theoretical framework of Deubel and Schneider (1996), these two forms of selection represent complementary components of a unified attentional control system. While Economides et al. (2014) demonstrated that attention can modulate which eye is used to initiate an action, our case shows that attention can determine which eye anchors perception when binocular fusion fails. Together, these findings highlight the flexible operation of visual attention across both perceptual and motor domains.

### Limitations

This study has several limitations: (a) The scoring of the patient's responses relied primarily on manual drawings, which are inherently subjective. (b) The analysis was based on only a few discrete two-dimensional magnetic resonance imaging slices rather than a full three-dimensional volumetric scan. (c) Although we inferred that those attentional mechanisms modulate the patient's reference eye, we did not directly manipulate or measure attention (e.g., through dual-task paradigms).

## Conclusions

In this study, we have clearly demonstrated that when binocular fusion fails, the human visual system relies on a single-eye reference frame across multiple viewing conditions. More remarkably, we found that this reference could be voluntarily switched: The patient was able to reassign spatial anchoring betweem the two eyes, regardless of stimulus shape similarity, stimulus exposure order, or participant response demands.
